# Incremental cost of increasing access to maternal health care services: perspectives from a demand and supply side intervention in Eastern Uganda

**DOI:** 10.1186/1478-7547-12-14

**Published:** 2014-06-19

**Authors:** Chrispus Mayora, Elizabeth Ekirapa-Kiracho, David Bishai, David H Peters, Olico Okui, Sebastian Olikira Baine

**Affiliations:** 1 Department of Health Policy Planning and Management, Makerere University School of Public Health, PO Box 7072, Kampala, Uganda; 2 Department of International Health, Johns Hopkins Bloomberg School of Public Health, 615 North Wolfe Street, 21205 Baltimore, MD, USA

**Keywords:** Vouchers, Maternal health, Costs, Sustainability, Demand-side, Supply-side

## Abstract

**Introduction:**

High maternal and infant mortality continue to be major challenges to the attainment of the Millennium Development Goals for many low and middle-income countries. There is now evidence that voucher initiatives can increase access to maternal health services. However, a dearth of knowledge exists on the cost implications of voucher schemes. This paper estimates the incremental costs of a demand and supply side intervention aimed at increasing access to maternal health care services.

**Methods:**

This costing study was part of a quasi-experimental voucher study conducted in two districts in Eastern Uganda to explore the impact of demand and supply - side incentives on increasing access to maternal health services. The provider’s perspective was used and the ingredients approach to costing was employed. Costs were based on market prices as recorded in program records. Total, unit, and incremental costs were calculated.

**Results:**

The estimated total financial cost of the intervention for the one year of implementation was US$525,472 (US$1 = 2200UgShs). The major cost drivers included costs for transport vouchers (35.3%), health system strengthening (29.2%) and vouchers for maternal health services (18.2%). The average cost of transport per woman to and from the health facility was US$4.6. The total incremental costs incurred on deliveries (excluding caesarean section) was US$317,157 and US$107,890 for post natal care (PNC). The incremental costs per additional delivery and PNC attendance were US$23.9 and US$7.6 respectively.

**Conclusion:**

Subsidizing maternal health care costs through demand and supply – side initiatives may not require significant amounts of resources contrary to what would be expected. With Uganda’s Gross Domestic Product (GDP) per capita of US$55` (2012), the incremental cost per additional delivery (US$23.9) represents about 5% of GDP per capita to save a mother and probably her new born. For many low income countries, this may not be affordable, yet reliance on donor funding is often not sustainable. Alternative ways of raising additional resources for health must be explored. These include; encouraging private investments in critical sectors such as rural transport, health service provision; mobilizing households to save financial resources for preparedness, and financial targeting for the most vulnerable.

## Introduction

High maternal and infant mortality continue to be major challenges to population health and the attainment of the Millennium Development Goals (MDGs) for many low and middle-income countries, including Uganda. A significant number of maternal deaths could be averted if timely access to skilled birth attendants and other quality maternal health care services were ensured [1]. There is now a vast amount of evidence on interventions that could address these challenges in many countries [2-4].

One of the interventions increasingly being used to increase access to maternal health services is maternal health service vouchers. These are demand-side vouchers that give pregnant women an opportunity to choose among several providers of care. The resulting competition among providers can lead to improved quality of care [5,6]. Using vouchers to subsidize health care services thus presents a shift away from the traditional input based to performance (output) based financing of health [1,5,7]. Vouchers have successfully been used to subsidize vulnerable populations to access critical health care services across many settings [8-17].

Often, however, many subsidy schemes are funded by donor agencies and continue to operate as small scale pilot schemes. In addition, such subsidy schemes generally suffer substantial administrative and operational costs. These costs present a challenge for sustainability, and scale-up. Furthermore, there is still paucity of information regarding their cost implications on health Cost information is essential for facilitating health policy and decision making [5,18].

### Background of the demand-side and supply-side intervention in Eastern Uganda

Despite several interventions, Uganda’s maternal mortality ratio and infant mortality rate are still high at 438/100,000, and 54/1000 respectively [19]. Factors that account for this include; limited access to supervised deliveries by trained health workers, inadequate emergency obstetric care, and the lack of timely appropriate postnatal care [20,21]. Studies have identified distance to health facilities, geographical inaccessibility of most rural areas worsened by poor transport and communication networks, and household socioeconomic factors as major constraints to accessing quality maternal health care services [22-24]. Statistics indicate that only 58% of deliveries were assisted by skilled birth attendants [19]. Remarkable inequalities in access also exist in terms of geographical location and household socioeconomic status. For example, only 36 per cent of women in the rural areas delivered in a health facility compared to 64 per cent in urban areas. Similarly women from the lowest socioeconomic quintile were less likely to deliver at a health facility (42 per cent) compared to those in the highest socioeconomic quintile (88 per cent) [19].

To investigate how to address constraints to accessing essential and quality maternal health services, a demand and supply side intervention was designed and piloted in two districts in Eastern Uganda in 2010–2011. This was part of a study undertaken through the Makerere University School of Public Health and Johns Hopkins University School of Public Health (MU - JHU) twinning program in collaboration with the Future Health Systems Research consortium (FHS-RPC) [25]. The designed intervention consisted of a voucher scheme (vouchers for transport and services), community mobilization, and health systems strengthening (including provision of basic supplies, training health workers, and support supervision).

The intervention was shown to produce a marked increase in ante-natal care (ANC), post-natal care (PNC) attendances and facility deliveries during the implementation period [26]. The findings showed that demand side financing arrangements that allow for use of locally available transport alternatives like (motorcycle and bicycle ambulances), combined with supply side initiatives (staff motivation and improving supplies) can potentially reduce barriers to access to quality maternal and newborn health care services [26,27].

In this paper, we estimate and present the incremental costs of this intervention, with a view of providing a better understanding of the additional costs and resources that would be required if such an intervention were to be integrated and implemented within the health care system.

## Methods

This costing study was part of a larger study whose implementation design details are extensively described in Ekirapa et al. [26]. Two districts in Eastern Uganda, namely; Kamuli and Pallisa, were included in this study. The districts were selected because they were comparable in terms of their poor maternal heath indicators. Both are rural (geographical location), and have limited capacity to offer maternal health services. Each district had three Health Sub districts (HSD) and one of these was randomly selected as an intervention HSD and one of the remaining two (which most closely reflected similar demographic composition and availability of health services infrastructure) was selected as a control HSD. A HSD is a health administrative area with population ranging from 30,000 to 100,000 with up to 10 health facilities.

In the intervention arm, transport and maternal care service vouchers were distributed to pregnant mothers at the ANC clinic during their first visit irrespective of the trimester of the pregnancy (Dec 2009 – March 2010 and June 2010 to June 2011). The transport voucher entitled a pregnant woman to obtain locally available transportation (motor cycle or bicycle) to and from an accredited health facility within their catchment area for four ANC visits, delivery and one PNC visit (hereafter called full package), while the service voucher entitled the pregnant mother to maternal health services at an accredited health facility of their choice. Eligible mothers received both transport and service vouchers. Upon utilizing the transport and maternal services, a mother submitted the voucher to the service provider (transporter or health worker). These service providers later presented the vouchers to the study team for cash reimbursement. Payments were made every three – four weeks as agreed with all service providers.

During the pilot period, a full package of services was provided (4 ANC sessions, delivery and one PNC session). In the initial period of the pilot, there was an unanticipated rise in demand for vouchers, and so the transport voucher costs increased. To ensure that these costs were contained, the service package to be covered was scaled down. Hence during the implementation, only delivery care and postnatal care services for those with complications were provided. The selection of delivery and PNC services was informed by available evidence that reveals that most maternal deaths occur during and soon after delivery [28]. However those who had received vouchers for the full package during the pilot period continued to receive all service entitlements including ANC. Women who were referred from a lower level facility to a higher level facility e.g., for a caesarean section or due to other pregnancy related complications, received a ‘special’ voucher for transport that entitled them to use other transport arrangements, usually public taxi or ambulance.

Although transport and service vouchers were provided only in the intervention arm, health facility strengthening (health worker training, provision of basic equipment and supplies, and support supervision) was done across both intervention and control arms.

**Figure 1:**
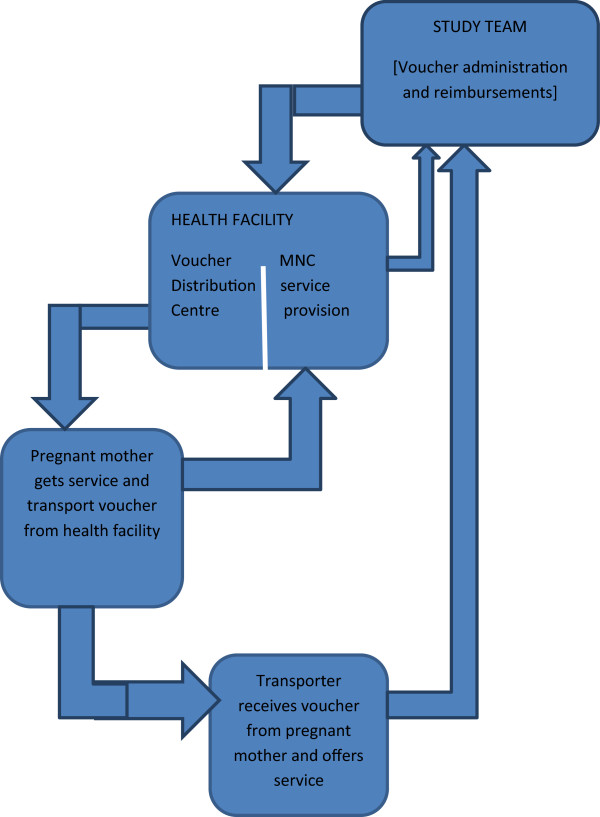
Design and implementation structure of the voucher scheme.

The study team from Makerere University School of Public health was responsible for the overall management and administration of the scheme. The team was headed by a study coordinator who liaised with transporters (through organized associations), the health facilities and the district health team. On behalf of the study team, the coordinator distributed vouchers to health facilities where mothers accessed them on their first ANC visits. Upon offering the service (transport and MHC), service provider’s submitted vouchers to the study team (coordinator) and upon verification, the coordinator effected their reimbursement.Figure 1, is a schematic presentation of the management and administrative structure of the voucher scheme that was part of the implemented demand and supply side intervention.

### Costs: sources, measurement and perspective

The costing analysis reported in this paper was done for a one year period (June 2010 to May 2011). Whereas research costs were not included, start-up costs for training and sensitization of communities and other stakeholders were included. For costs that were incurred in the previous year, adjustments were made for inflation. Program costs were analyzed using ingredients approach to costing [29]. Costs were estimated from the provider’s perspective and measured retrospectively based on project accounting, financial and administrative records. The objective of focusing on only the provider perspective was to allow for an understanding of the cost implications of such an intervention in terms of any additional burden to a national health care budget. Thus, economic costs and user contributions are excluded from this analysis. Vouchers were valued according to the reimbursement agreements made between the program and transport and health service providers. Other procurements for the program such as basic clinical supplies distributed across health facilities were valued according to market prices. Costs were classified according to major activity and or source. The different activities and all inputs used for those activities in the intervention area during the implementation period of the scheme were identified and respective costs obtained.

### Voucher costs

The voucher costs included in this study reflect the rates competitively negotiated by the study team and the transport and health service providers. Transport voucher rates ranged between 0.9 USD and 2.3 USD, depending on how far the facility was located, and fuel prices at the time. Payment rates for the public and private facilities were different. The public health facilities received 75 per cent of the rates paid to Private-not-for Profit (PNFP) facilities (Table 1). This is because public facilities are fully financed by government, while the PNFPs receive only partial government support or no support at all. Payment rates for service vouchers also varied during the implementation period. This variation was due to the changing market conditions for the service inputs (supplies), and the initial recorded unanticipated surge in demand for the vouchers. Table 1 provides details of the service voucher fee schedules and fee changes over the implementation period.

**Table 1:** Fee schedule of health services for different regimes (US$)

**Service**	**Pilot phase Dec, 2009-May, 2010**		**Implementation period June, 2010-Feb, 2011**		**Implementation period March, 2011-Dec, 2011**	
	**Gov’t**	**PNFP**	**Gov’t**	**PNFP**	**Gov’t**	**PNFP**
**ANC1**	0.9	1.1	0.4	0.55	0.4	0.55
**ANC 2,3,4**	1.0	1.4	0.4	0.55	0.4	0.55
**Delivery**	5.1	6.7	2.7	3.64	4.1	5.45
**C-section**	51.1	68.2	29.5	59.1	27.3	54.5
**PNC**	1.0	1.4	0.4	0.55	0.55	0.55

US$1 = 2,200Ug.Shs.

To avoid duplication, forgery and possible fraud, the vouchers contained key client identification information (serialized) that was also recorded in the voucher book and the facility registers. These records were used as the basis for reimbursement for maternal health service vouchers after verification for authenticity.

### Health system strengthening

Health facility strengthening costs were associated with training of health workers at health facilities, procurement and distribution of basic drugs, equipment and other supplies, as well as provision of support supervision. Health system strengthening was conducted in both the control and intervention areas for both public and PNFP facilities. These costs were captured separately for the intervention area and control area. In this paper, we report costs for health system strengthening in both areas.

### Voucher scheme administration costs

Administration costs for the scheme were estimated through reviews of project documents. Administration costs related to expenditures on personnel such as field coordinators and field supervisors. Other costs here included transport for regular monitoring, supervision, coordination, communication, and data collection. Expenditures on other overhead costs such as space, management and finance support were excluded since they were not considered to have been core to the running of the program and their omission could not make a significant difference.

### Estimating total costs, unit costs, and incremental costs

Costs incurred during the voucher scheme implementation consisted of both fixed costs and variable costs. Fixed costs included those incurred on administration, sensitization, mobilization, procurement of equipment, and supervision of health facilities. On the other hand, variable costs depended on the number of women utilizing the services. These included cost of transport vouchers, service vouchers, and drugs and supplies. Costs for additional utilization were not calculated separately for two main reasons. It was assumed that costs for additional supplies and equipment which could have been required as a result of the increased utilization had been captured in the costs for the additional supplies and equipment which were supplied as part of the intervention. On the other hand, additional time spent by health workers, was compensated through allowances provided through the service vouchers, implying that these costs are already counted under the service voucher costs. However it is important to mention that the costs could have been underestimated since we did not directly calculate the increase in inputs.

### Unit costs

Unit costs were estimated for each service provided. This was measured as the total costs per service divided by the number of service users. We separately estimated the average transport costs for each service (for example average total costs for deliveries, ANC and PNC). This was estimated by dividing the total costs incurred in transporting mothers by the number of women transported for these services. Note that there is an overlap for women who received all three services. The data available could not allow us to segregate such women. Women who underwent caesarian section were often referred to a higher level hospital usually outside the catchment area. In such cases, costs for referral transport were higher than the costs for routine transport, and so it was not appropriate to calculate an average cost that would combine routine and referral transport. The authors excluded these in this calculation.

Incremental costs for delivery and PNC were later obtained by estimating the additional services induced bythe intervention program. These estimates of incremental effects were obtained by calculating the difference in service utilization (institutional deliveries and PNC) one year prior to the program implementation (December 2008 – November 2009), and during the one year of implementation (June 2010 – May 2011) for both control and intervention areas. The incremental services induced in the intervention and control areas were then added together. These incremental effects were adjusted for underlying service utilization trends in the country based on data from the Uganda Demographic and Health Survey [30] that estimated a 3% annual increase in institutional deliveries from the year 2005 to 2010. The final estimation is based on the assumption that after adjusting for secular trends, the difference in utilization is attributed to the intervention.

To understand whether there were other factors that could have influenced the observed difference in service utilization, we collected information on other maternal and child health (MCH) programs in the program area. We found evidence of two other programs (irregular supply of birth kits by the government and provision of ultra sound services in one health facility). Our assessment showed that they were not likely to explain the changes in MCH service utilization observed.

### Incremental costs

Incremental costs in this context were defined as the additional costs that a health system would incur in order to achieve additional utilization (through increased access) of maternal services, beyond what the system currently provides. The additional costs for each service utilized were obtained by attributing major activity costs to each service, based on the proportion of patients who utilized the service.

## Results

### Service Utilization

A total of 22 lower level public and private facilities and 3 referral hospitals, all offering antenatal, delivery and postnatal care services, were involved in this program. The three referral hospitals mainly provided caesarian sections, evacuations, and occasionally normal deliveries for mothers who had been referred for a caesarian section but succeeded in delivering normally. During the implementation period (June 2010-May 2011), a total of 39,348 mothers were transported to and from the health facilities for ANC, delivery and PNC in the intervention area. This number was based on records from reimbursed service vouchers available in the health facility records (Table 2). The number of additional deliveries conducted during the implementation period was 13,283 while additional PNC attendances were 13,780, after a 3% adjustment for the underlying trends. This data was based on the health facility MCH service utilization registers. From these registers, baseline statistics for PNC were very low, and this partly accounts for the high additional PNC attendances recorded. More details about changes in service utilization due to the voucher scheme are available in Ekirapa et al. [26].

**Table 2:** Service vouchers reimbursed in the intervention period

**Service**	**Total**	**Percentage of total**
**ANC**	6302	15.9
**Deliveries**	22558	57.0
**PNC**	9678	24.4
**C-Section**	213	0.5
**False labour**	810	2.0

### Total, average, and incremental costs

Using the provider’s perspective, the estimated financial cost of the intervention including the voucher scheme for the one year implementation period was US$525,472 (at the exchange rate of $1 = 2200UgShs, and 2010 prices). Transport voucher costs (35.3%), health system strengthening costs (29.2%), and service voucher costs (18.2%), formed a significant portion of the total financial costs. Table 3 below, presents major cost drivers for the intervention.

**Table 3:** Distribution of costs by major expenditure

**Activity cost**	**Amount in US$**	**Percentage**
**Service voucher costs**	95,866	18.2
**Transport voucher costs**	185,376	35.3
**Health systems strengthening**	153,028	29.2
**Sensitization and mobilization**	41,334	7.9
**Administration**	49,869	9.5
**Total**	**525**,**472**	**100.00**

Other costs included sensitization and mobilization (7.9%), and central support costs including administration (9.5%). A detailed description of the subcomponent costs within these broader cost centres can be found in Table 4 below.

**Table 4:** Detailed cost expenditure lines under major components

**Activity**	**Amount US$**	**Total amount US$**
Transport vouchers reimbursements	174,666	185,376
Admin costs	4,793	
Contingency	482	
Printing vouchers	3,809	
Identification cards	1,622	
Service vouchers reimbursements	87,680	95,866
Admin costs for payments	4,135	
Contingency	241	
Printing vouchers	3,809	
Equipment, drugs & sundries	101,822	153,028
Support supervision	13,917	
Training of health workers	36,673	
Training materials	616	
**Total health system strengthening**		

The voucher reimbursement costs reported in this study were US$0.55 for ANC, US$3.6 for deliveries; US$0.55 for PNC and US$28 for C-section in public facilities. Similarly, normal deliveries and C-sections in PNFPs were reported at an average US$4.5 and US$52 respectively (Table 1).

Given the reported number of women transported during the implementation period for ANC, delivery or PNC, and the total amount spent on transport voucher reimbursements (US$185,376), the estimated average cost for transport per woman transported, excluding referral transport, was US$4.6. The total incremental costs incurred on deliveries (excluding caesarean section) amounted to US$317, 157 and US$107,890 for PNC. These costs combine payment for both transport and service vouchers for all ANC sessions (for those who had full package) and delivery, health system strengthening, sensitization and mobilization, as well as voucher administration. These costs were obtained by apportioning the total costs incurred in all cases to deliveries and PNC according to the number of women who utilized those services (58% and 24% respectively). Based on this, the estimated incremental cost per additional delivery was US$23.9, and US$7.9 per additional PNC attendance (Table 5).

**Table 5:** Incremental costs for delivery and postnatal care

**Item**	**Incremental cost for delivery (USD)**	**Incremental cost for delivery PNC (USD)**
Service vouchers	73296.4	3267.0
Transport component	104598.8	44,875.7
Health System	87258.2	37,436.2
Sensitization	23568.3	10,111.0
Administration	28435.5	12,199.6
Total	317157.1	107,890.1

## Discussion

Although there is evidence from various studies that demand side interventions such as using vouchers help to increase the utilization of maternal health services [10-14], there is limited information about their costs, feasibility and sustainability. This study estimates cost per additional delivery as US$23.9 and US$7.9 for PNC. The average reimbursement costs for ANC, deliveries; PNC and C-section were on average: US$0.55, US$3.6, US$0.55 and US$28 for public sector (average US$52 for C-section in PNFP) respectively. A similar study in Pakistan reported reimbursement costs to providers to be US$1.25 for ANC and PNC, US$31 for delivery US$125 for C-section. These costs were much higher than reported in this study. This variation may be explained by local differences such as the cost of supplies and inputs, as well as differences in the type of technology. These contextual differences often make comparability of voucher costs across countries difficult to achieve. When compared to the costs for another voucher program implemented in Uganda in relatively similar contexts, we find costs for this study comparably lower [31]. A full cost effectiveness study for this intervention [32] found an incremental cost-effectiveness ratio (ICER) of US$302 per disability adjusted life years (DALY) averted compared with the status quo. The study concluded that using vouchers was a cost-effective approach to increasing access to maternal health care services particularly for the poor.

According to our findings, the main cost drivers for maternal care were the transport voucher reimbursement (35% of the costs), and health system strengthening (29.2% of the costs). Indeed several studies have echoed the need to ensure access to transport services as an important element to increasing accessibility to maternal health services. The average cost for transport in this study was comparable to what was reported in the Maternal Health Voucher Scheme (MHVS) in Bangladesh [11] and the Pakistan study [13]. One possible way to overcome the high costs of transport could be for government to extend obstetric care provision to the communities by integrating obstetric care into services provided at lower level health centres located within the communities. This would also require improving their capacity to provide such care, including recruiting trained personnel, and acquiring relevant and up to date medical equipment and technologies [33].

As noted above, the costs for health system strengthening initiatives were one of the major cost drivers. Indeed, improvements in the quality of services are required if utilization of services is to increase. Poor quality services are one of the reasons reported as contributing to low utilization of maternal health services in the developing world [34,35]. Whereas the government of Uganda has recently embarked on expanding health infrastructure and increasing the presence of health workers in rural areas in a bid to address the quality issues, persistent stock-outs of essential medicines, supplies and absence of medical equipment is still apparent [36]. Therefore, investment in increasing the quality of services is necessary. One of the costs that were not assessed in this study was the cost of the increased workload that results from increased demand for services. In very busy facilities, the Ministry of Health may be required to recruit more personnel, to match the increased service utilization that may result from such interventions.

The lowest costs were incurred on community sensitization and mobilization (7.9%) and administration (9.5%). Other voucher programmes have commonly been managed by voucher management agencies, a system that often leads to very high administrative costs. The main administrative costs in this study were incurred during payment of the transport and service vouchers. Administrative efficiency was achieved by arranging for program monitoring to be done together with the district health office as part of its routine mandate.

An outstanding question, that this and other similar studies have not addressed, is related to how such schemes could be sustained using locally available resources. Many voucher schemes are often piloted or even implemented using donor funds. From this study, the additional cost per additional delivery is $23.9. This is more than two times the public health expenditure per capita of US$10 for Uganda [37], and represents 58% of the total per capita expenditure on health which is US$41. Clearly, current public sector allocations to health may not sustain such a scheme, if it were to be rolled out. To be feasible, government would either have to increase its allocation to the health sector or leverage additional resources from the private sector. This could be through creating incentives that enhance public-private partnerships for health, and private investments in critical sectors such as rural transport system, and health service provision. In addition, households and individuals could be mobilized to save financial resources through locally organized and managed pools, from which funds could then be drawn and used to provide transport for routine and emergency maternal health services for the members of the pool. In Uganda for example, structures for microfinance such as Savings and Credit Cooperative Organizations (SACCOs) and other women groups already exist within communities. These could be taken advantage of in this regard. Encouraging such community savings initiatives offers an additional advantage, namely; providing an experience of prepayment - valuable for promoting participation and enrolment into the national health insurance scheme that is currently under discussion in Uganda.

Both this paper and Alfonso et al. [32] draw from the same dataset to answer different costing questions, and indeed find different results. For example, whereas this paper reports the total costs of the program as US$525,472, Alfonso et al. finds the total cost as $314,316. These differences are attributed to the fact that this paper included costs of both the control and intervention areas, in addition to ANC & PNC related costs. Other variations in costs could be attributed to annualization of capital costs done by Alfonso et al. This paper did not annualize the costs for any fixed inputs (a possible limitation), because the authors aimed at estimating the initial resources that government would require if it were to implement such a program, irrespective of when benefits would accrue. Finally, whereas Alfonso et al. calculates the average cost per delivery ($19.65) by considering costs of all deliveries in the intervention area, this paper calculates the average cost per additional delivery ($23.9) by considering additional costs and effects (deliveries) incurred in both intervention and control areas and additional deliveries from both arms.

## Conclusion

Subsidizing maternal health care costs through demand and supply – side initiatives may not require significant amounts of resources contrary to what would be expected. With Uganda’s Gross Domestic Product (GDP) per capita of US$551 (2012). This is about 5% of GDP per capita to save a mother and probably her new born. This level of subsidy may not be affordable for most low income countries, given their limited resource envelops, yet reliance on donor funding is often not sustainable. Alternative ways of raising additional resources for maternal health should be explored, including encouraging private investments in critical sectors such as rural transport and health service provision. Households should also be mobilized to engage in income generating activities so as to raise and save financial resources, that could then be used to cushion them during emergency care seeking. The limited government resources could then be targeted towards providing services for the most vulnerable who may not be able to make the required financial contributions. In addition, focusing on subsidizing costs for maternal services may not be the panacea to realizing better maternal and child health outcomes. Initiatives that improve and safeguard the quality of services are also important for achieving better maternal health outcomes, including health workers' training, support supervision, provision of essential supplies, among others.

We however, recommend that more studies should be undertaken on the cost-effectiveness of demand and supply side subsidy initiatives in improving maternal health outcomes especially in the developing countries and how they can be sustained with minimal reliance on donor funding. Further investigations should also include a focus on implications in terms of extra workload for health workers that a subsidy scheme might generate. This would aid human resource planning over the medium and long-term.

## Competing interests

The authors do not have any competing interests in this paper.

## Authors’ contributions

CM, EEK, contributed to the design of the study and co-wrote drafts of the manuscript. DHP and SOB also contributed to the formulation of the study and provided substantial input into the manuscript. DB and OO reviewed and provided substantial input into the manuscript. All the authors read, provided substantial input and approved the final manuscript.
